# Frozen-Thawed Sperm Analysis of Benign Prostatic Hyperplasia Dogs Treated With Finasteride

**DOI:** 10.3389/fvets.2022.901943

**Published:** 2022-06-30

**Authors:** Renato Bueno Flores, Daniel de Souza Ramos Angrimani, Maira Morales Brito, Leticia Lima de Almeida, João Vitor Menezes Lopes, João Diego de Agostini Losano, Camila Infantosi Vannucchi

**Affiliations:** Department of Animal Reproduction, School of Veterinary Medicine and Animal Science, University of São Paulo, São Paulo, Brazil

**Keywords:** canine, cryoinjury, finasteride, treatment, Benign Prostatic Hyperplasia

## Abstract

Benign Prostatic Hyperplasia (BPH) is a pathological condition that directly interferes with the reproductive potential of senile dogs, by leading to prostate enlargement and sperm injury, which in turn may compromise sperm freezeability. Moreover, albeit finasteride treatment reduces prostatic volume and blood supply and maintains seminal quality and testicular integrity, the effects of sperm samples submitted to cryopreservation after the finasteride treatment are still unknown. Thus, the aim of this study was to evaluate frozen-thawed semen of BPH dogs, as well as dogs subjected to BPH pharmacological treatment with finasteride. For such purpose, 20 dogs were previously selected and assigned to three experimental groups, according to BPH diagnosis and treatment with finasteride: Control (*n* = 9), BPH Group (*n* = 5) and BPH-Finasteride Group (*n* = 6). Semen was subjected to one-step cryopreservation protocol with tris-fructose-citric acid extender with 5% glycerol and thawed at 37°C for 30 sec. Fresh and post-thaw sperm samples were evaluated for macroscopic parameters, sperm concentration, sperm motility kinetics, sperm mitochondrial activity and potential, oxidative stress, plasmatic and acrosome membrane integrity, sperm DNA fragmentation and sperm binding test on perivitelic membrane of chicken egg yolk. Regarding fresh semen, BPH-Finasteride group had the lowest ejaculate visual aspect (opacity), higher frequency of sperm flagellar beating (BCF) and percentage of sperm with medium velocity. Control group had the highest percentage of sperm DNA integrity compared to BPH group. For the frozen-thawed semen, Control group presented the highest percentage of spermatozoa with high mitochondrial activity. However, the BPH-Finasteride group showed higher number of sperm bound to the perivitelline membrane of chicken egg yolk compared to the BPH Group. Conversely, BPH group had higher percentage of DNA damage. In conclusion, the ejaculate of BPH dogs has higher susceptibility to cryoinjury, whereas finasteride-treated dogs have increased spermatozoa functional performance, suggesting a promising use of BPH dogs as semen donors in sperm cryopreservation programs.

## Introduction

Nowadays, canine geriatric patients are referred to assisted reproduction services aiming to achieve a better reproductive performance, since several aging diseases directly affect their reproductive potential (1). In this respect, Benign Prostatic Hyperplasia (BPH) is a highly frequent reproductive disease of elderly, non-castrated dogs, negatively affecting male reproductive performance (2–5).

In a reproductive point of view, BPH is marked by prostate enlargement with significant sperm alterations, such as DNA fragmentation (3, 6), motility kinetics (2), oxidative stress status (5) and high percentage of sperm defects (2). The causes under BPH altered sperm features are related to a remarkable hormonal imbalance, affecting both spermatogenesis and epididymal maturation (7, 8). Moreover, biochemical alterations of the prostatic fluid (p.e., hematospermia) lead to an incompatible milieu for sperm osmolarity and stability (9–11). Such seminal physical-chemical alterations are undesirable for sperm reproductive biotechnologies, as for example, cryopreservation (12). However, the effect of BPH on sperm freezability and cryoinjury on seminal samples previously compromised are still unknown.

For the medical treatment of BPH, drugs that reduce prostatic enlargement and hormonal imbalance are preconized. For such purpose, finasteride is the drug of choice for breeding dogs, by leading to reduction in prostatic volume and blood supply (6) and maintaining semen quality and testicular integrity even after two months of treatment (2, 13). Finasteride is a 5α-reductase inhibitor, which decreases 70% of circulating dihydrotestosterone (DHT) levels (14). DHT formation is catalyzed by the enzyme 5α-reductase, which is a protease with two isoenzymes that can convert testosterone to DHT and accelerate the progression of prostate hyperplasia, growth and progression of BPH. Whenever finasteride treatment is discontinued, DHT levels increase progressively again in 2 weeks (15). Locally in the prostate, 5α-reductase inhibitors downregulates concentration of DHT by 90%, as finasteride is a competitive inhibitor of types II and III isoform of 5α-reductase (3).

BPH medical treatment allows for the use of stud dogs in reproductive biotechnologies, such as artificial insemination and sperm cryopreservation, although the effect of finasteride treatment has not been yet investigated in dogs.

Thus, this study aimed to evaluate frozen-thawed semen of BPH dogs, as well as dogs subjected to BPH pharmacological treatment with finasteride.

## Materials and Methods

### Ethics Committee

The present study was previously approved by the Bioethics Committee of the School of Veterinary Medicine and Animal Science – University of São Paulo (protocol number 5019100217).

### Experimental Design

This experiment was designed as to evaluate sperm samples before and after cryopreservation, i.e., both fresh and frozen-thawed semen of the same subject were analyzed.

Twenty senile male dogs of varied breeds and body weights (average of 18.8 kg), between 6 and 13 years old (average 10.6 years) were used. Because the body mass index may influence sperm quality and senility, the body weight of each dog was taken into account as an inclusion creteria, i.e., small dogs were considered senile with more than 8 years, medium dogs with more than 7 years old and large dogs with more than 6 years (16).

Dogs were assigned to three experimental groups according to BPH diagnosis and finasteride treatment:

**BPH group** (*n* = 5, mean age 9 ± 2.8 years and body weight 20.62 ± 13.9 kg): dogs with the presumptive diagnosis of BPH, which was based on clinical signs, prostatic secretion assessment and prostatic biometry by B-mode ultrasound (4). The most common clinical signs of BPH were prostatomegaly coupled with hematospermia, hematuria, dysuria and tenesmus. Only dogs that presented hematospermia and at least one general clinical sign (tenesmus, hematuria or dysuria) were considered BPH dogs;

**BPH-Finasteride group** (n = 6, mean age 12 ± 2.5 years and body weight 18.78 ± 11.4 kg): dogs that were diagnosed presumptively with BPH were subsequently subjected to oral finasteride therapy (5 mg of finasteride per dog daily - Finasterida®/Medley®) during 60 days (17);

**Control group** (n = 9, mean age 8 ± 2.1 years and body weight of 23.77 ± 9.8 kg): dogs without prostatomegaly or any other clinical signs of BPH.

To assure the appropriate sample size, an analysis was conducted with the SAS Power and Sample Size 12 (SAS Institute Inc., Cary, NC, EUA). A retrospective analysis of the percentage of post-thaw sperm DNA integrity, acrosome membrane injury and minor sperm defects indicated there was a power of >0.99, which is considered an acceptable statistical power (at least 0.8). Hence, a minimum of five dogs per group were sufficient to demonstrate significant differences in the data.

Before onset of the experiment, each dog was subjected to a through breeding soundness exam, which allowed not only the diagnosis of BPH, but also additional renewal of sperm reservoir. For the purpose of the present experiment, a single sperm sample was obtained from each male and only the sperm-rich fraction of the ejaculates was collected by digital manipulation of the penis. The first fraction of the ejaculate was discarded and the sperm-rich fraction was collected directly into calibrated plastic tube through plastic funnels. Care was taken to separately collect the second fraction of the ejaculate, by means of the color aspect of the ejaculate.

### Cryopreservation Protocol

After the collection, samples were centrifuged at 200 × g for 5 min in room temperature and the supernatant was discarded. We used an own production tris-fructose-citric acid extender (0.26 M tris-hydroxymethyl- aminomethane, 0.14 M citric acid monohydrate, 0.06 M D-fructose, 20% egg yolk, 0.02 M of gentamicin and distilled water, with 5% glycerol, 1,179 mOsm/L, pH 6.95), previously described by Brito et al. (10). The pellet was resuspended in the extender at 37°C, in a final sperm concentration of 200 million sperm/mL. Diluted samples were placed into a refrigerator at 5°C inside a beaker containing water. Samples were refrigerated using a slow cooling curve until they reached 5°C, keeping control of the cooling curve with a thermometer. After this period, semen was packaged in 0.5 mL straws, kept in nitrogen vapor for 20 min and sequentially immersed and stored in liquid nitrogen (10).

### Fresh and Post Thawed Semen Evaluation

Immediately after collection, the volume of the sperm-rich fraction was measured in a graded test tube (mL), in addition to an arbitrary scale of the sample visual aspect of opacity, graded as 1 to 3: 1- translucid sample; 2 – moderated opacity; and 3 – high opacity. Sperm count was performed using a haemocytometer counting chamber under a 1:200 dilution in a methylene blue solution with an optical light microscope at 400 × (Nikon, Eclipse E200, Japan) and the total sperm count per sperm-rich fraction of the ejaculate was calculated (18).

For both the fresh and post-thawed samples, sperm kinetics was evaluated using the Computer Assisted Sperm Analysis (CASA; HTM-IVOS Ultimate 12.3; Hamilton Torne Biosciences, Beverly, MA, USA) according to the protocol described for dogs (19). Briefly, an aliquot of 7 μL was deposited between a slide and cover slip previously heated to 37°C. Then, 10 fields were randomly selected and evaluated for the following parameters: motility (%), progressive motility (PROG, %), average pathway velocity (VAP, μm/s), straight-line velocity (VSL, μm/s), curvilinear velocity (VCL, μm/s), amplitude of lateral head displacement (ALH, μm), beat cross frequency (BCF, Hz), straightness (STR, %) and linearity (LIN, %). Based on the speed of sperm movement, spermatozoa was classified into four different groups: rapid (RAP, VAP>50 μm/s, %), medium (MED, 30 μm/s<VAP<50μm/s, %), slow (SLOW, VAP<30μm/s or VSL<15μm/s, %) and static (STATIC,%). Moreover, sperm samples were evaluated for conventional sperm progressive motility (%) and velocity (arbitrary scale from 0 to 5) using a drop (5 μL) of semen placed onto a pre-warmed glass slide with coverslip. Evaluation was performed under light microscopy (Nikon, Eclipse E200, Tokyo, Japan) at 400×magnification.

For the evaluation of sperm morphology, samples were diluted in saline formaldehyde solution at 1:2 ratio. Subsequently, 5 μL of the solution was placed between a slide and a coverslip and analyzed under phase contrast microscopy (Nikon, Eclipse E200, Japan) by counting 100 sperm cells. Sperm morphological abnormalities were classified as major (any abnormality related to infertility or pathological conditions in the testes or epididymides) and minor (any abnormality recognized as less important), according to Barth and Oko (20).

Sperm membrane integrity was analyzed by eosin/nigrosine stain (21), which revealed the percentage of live/dead spermatozoa. In brief, 5 μL of semen and 5 μL of the previously prepared stain were placed in a pre-warmed glass slide. Smears were evaluated at 1000× magnification under a light microscope (Nikon, Eclipse E200, Japan) counting 100 cells per slide. We considered damaged sperm (membrane lesion) as pink colored cells, while intact sperm (membrane integrity) presented no stain.

For acrosome membrane integrity assessment, we used the fast green/rose bengal stain, as described by Pope et al., (22), by mixing 5 μL of semen and 5 μL of the stain solution for 60 sec. Subsequently, smeared slides were analyzed at 1000× magnification under a light microscope (Nikon, Eclipse E200, Japan) counting 100 cells per slide.

Sperm mitochondrial activity was assessed based on the oxidation of 3'3 diaminobenzidine (DAB) by cytochrome-C (23). Briefly, 25 μL of semen were incubated with 25 μL of DAB solution (1 mg/mL in PBS, pH 7.0) in an amber microtube for 1 h at 37°C. Then, mixture was smeared on microscopy slides protected from light and fixed in formaldehyde (10%) for 15 min. Analysis was performed under light microscopy (Nikon, Eclipse E200, Japan) in a magnification of 1,000×. A total of 100 sperm cells were evaluated and classified into four classes: high mitochondrial activity (%), medium mitochondrial activity (%), low mitochondrial activity (%) and absence of mitochondrial activity (%).

In order to assess sperm DNA integrity, the modified toluidine blue stain technique was used (24). Sperm smears were prepared using 10 μL of semen samples and then fixed in 96% ethanol–acetone for 30 min at 4°C. After drying, smears were then hydrolysed in 0.1 N HCl for 5 min at 4°C and washed three times in distilled water for 2 min each. Subsequently, smears were exposed to toluidine blue stain (0.05%) for 20 min and then washed two times in distilled water for 2 min each. Smears were evaluated under light microscopy (Leitz, Dialux 20, Germany) at 1,000× magnification. The percentage of stained sperm was analyzed by counting 200 cells.

In order to analyze the integrity of sperm plasma and acrosome membrane, semen were analyzed by flow cytometry using the BD FACSCalibur equipment (Becton Dickinson, East Rutherford, NJ, USA). Sperm cells were previously stained with fluorescent probes according to the specific analysis, using a fixed sperm concentration of 188,000 cells diluted in 37.5 μL of TALP (Tyrode‘s Albumin Lactate and Pyruvate). For the analysis of plasma and acrossomal membrane integrity, propidium iodide fluorescent probe (PI; 6 μM in TALP) was combined with *Psyllium agglutinin* conjugated with fluorescence isothiocyanate (FITC-PSA; 100 μg/mL of 1% sodium azide). The mixed probe solution was added to diluted sperm and incubated at 37°C for 5 min. Subsequently, 300 μL of TALP was added and analyzed by flow cytometry, according to the protocol previously described (2). For the analysis of sperm mitochondrial potential, 1 μL of 50 μg/mL JC-1 probe (5,5', 6,6' tetrachloro-1,1,3,3'-tetraethylbenzimidazolylcarbocyanine iodide) was added to diluted sperm. Samples were incubated at 37°C for 10 min and then 300 μL of TALP were added and analyzed by flow cytometry, according to protocol previously described (2).

For the sperm binding test in perivitelline membrane of chicken egg yolk, a previously validated protocol for dogs was used (10). For this, segments of egg yolk perivitelline membrane (0.5 cm^2^) were transferred to petri dish containing PBS (phosphate-buffered saline), after several washes, it was removed the excess of yolk and the membrane became translucent. Then, membranes were incubated with 50 × 10^3^ sperm cells with canine capacitation medium (10) at 37°C for 1 h. After incubation, membranes were washed in a macrocentrifuge with tubes containing 10 mL of PBS to remove sperm not bound to the membrane. Subsequently, membranes were observed using a slide and cover slip to count the cells attached, it was used a phase contrast microscope with 400× magnification. Spermatozoa were counted in 3–5 fields and results were expressed in percentage of spermatozoa fixed by mm^2^ in the perivitelline membrane.

### Oxidative Status Assessment

The analysis of sperm susceptibility to oxidative stress was performed by determining the concentration of substances reactive to thiobarbituric acid (TBARS) after induced lipid peroxidation. TBARS test was performed according to protocol described by Ohkawa et al. (25), in which 100 μL of ferrous sulfate (4 mM) and 100 μL of sodium ascorbate (20 mM) were incubated for 90 min (37°C) with 400 μL of the sample. Subsequently, 1,000 μL of 10% trichloroacetic acid in 500 μL of this mixture (2:1 ratio) was added. Samples were centrifuged (18,000g, for 15 min at 15°C) and the recovered supernatant (500 μL) was transferred to glass tubes containing 500 μL of 1% (v/v) thiobarbituric acid in 0.05 N sodium hydroxide. Subsequently, samples were subjected to a temperature of 100°C for 20 min. After incubation, reaction was blocked by thermal shock, submitting the samples to a 0°C cold bath. The thiobarbituric acid reactive substances (TBARS) were quantified spectrophotometrically at a wavelength of 532 nm (Ultrospec 3,300 Pro, Amersham Biosciences, Little Chalfont, UK). The lipid-peroxidation index was described as nanograms of TBARS/10^6^ sperm. Results were compared to a standard curve previously prepared with a standard solution of malondialdehyde.

### Statistical Analysis

All data were evaluated using SAS System for Windows (SAS Institute Inc., Cary, NC, USA). Variables were tested for normality of the residues (normal distribution) and homogeneity of the variances. Whenever one of these assumptions was not respected, data were transformed. All variables were parametric, except for the percentage of major sperm defects, low sperm mitochondrial potential, acrosome membrane injury and plasma membrane injury, which were log transformed.

The ANOVA test and the Least Significant Differences (LSD) were used to compare the experimental groups in each moment (fresh and post-thawed samples). Results were described as untransformed means ± SE. The significance level used was 5%.

## Results

**Figure 1:**
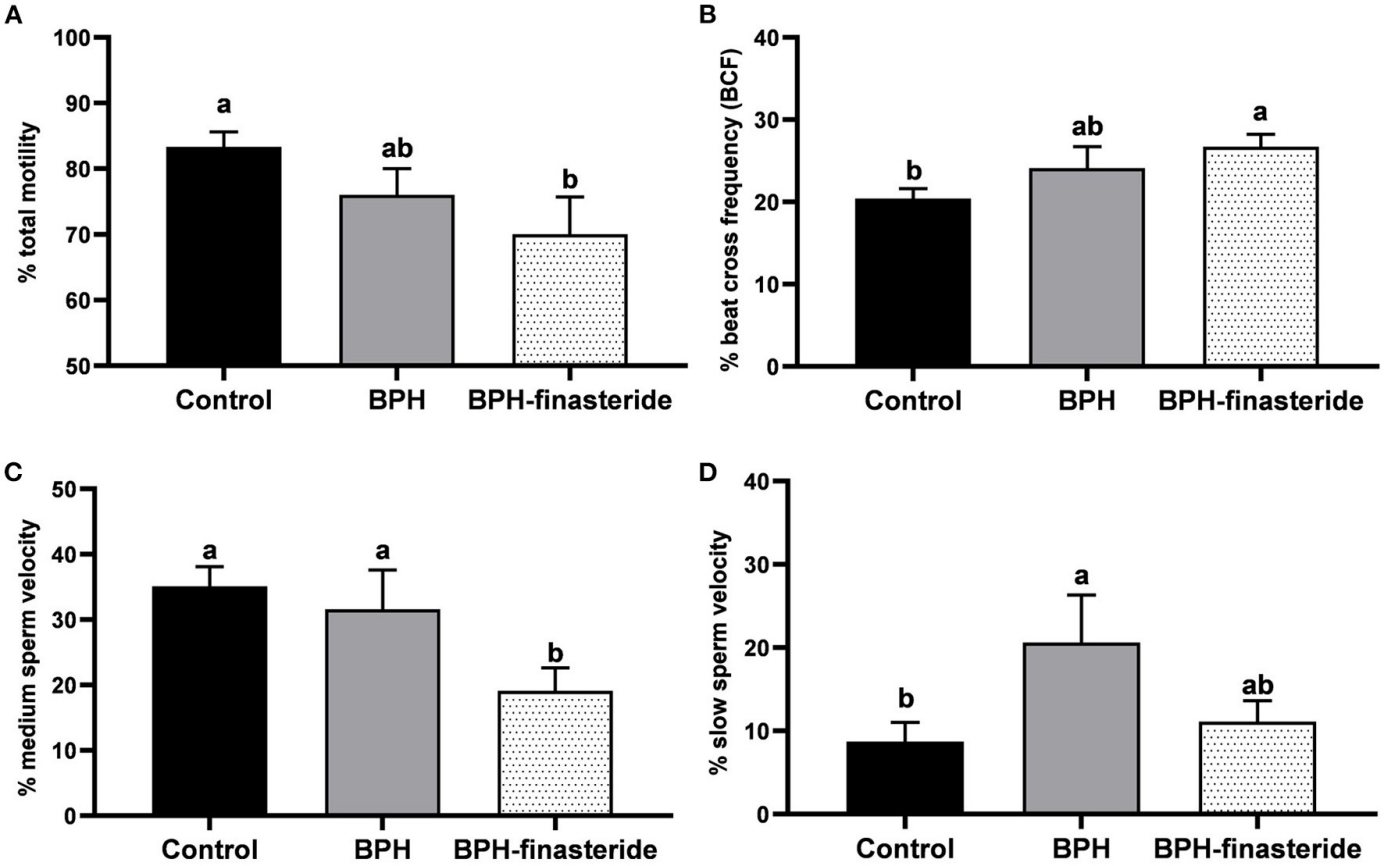
Mean and standard error of the fresh **(A)** sperm total motility (%); **(B)** sperm beat cross frequency (%); **(C)** medium sperm velocity (%) and **(D)** slow sperm velocity (%) in the Control, BPH and BPH-Finasteride groups. ^a−b^ indicate significant difference (*P* < 0.05) among experimental groups.

For the fresh samples, the Control group had higher sperm total motility in comparison to the BPH-Finasteride group (Figure 1A). On the other hand, the BPH-Finasteride group had greater sperm beat cross frequency compared to Control dogs (Figure 1B). The BPH-Finasteride group had the lowest (19.1 ± 3.5%) medium sperm velocity, in comparison to the Control (35.1 ± 3%) and BPH (31.6 ± 6%) groups (Figure 1C), whilst the percentage of slow sperm velocity was higher in BPH group compared to the Control Group (Figure 1D).

**Figure 2:**
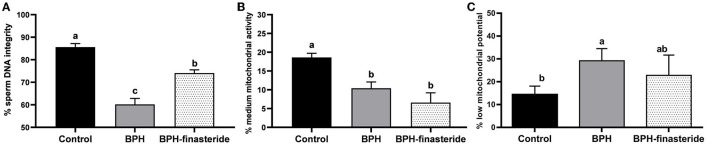
Mean and standard error of the fresh **(A)** sperm DNA integrity (%); **(B)** sperm medium mitochondrial activity (%) and **(C)** sperm low mitochondrial potential (%) in the Control, BPH and BPH-Finasteride groups. ^a−b^ indicate significant difference (*P* < 0.05) among experimental groups.

The BPH-Finasteride group had the lowest (*P* < 0.05) ejaculate aspect of opacity (1.6 ± 0.3), compared to the BPH (2.8 ± 0.2) and control groups (2.7 ± 0.1). The DNA integrity rate was higher in the Control group compared to the BPH-Finasteride group, which in turn was superior to the BPH group (Figure 2A). The Control group had the highest percentage of spermatozoa with medium mitochondrial activity (Figure 2B). On the other hand, the BPH group had higher percentage of spermatozoa with low mitochondrial potential in comparison to the Control group (Figure 2C).

**Figure 3:**
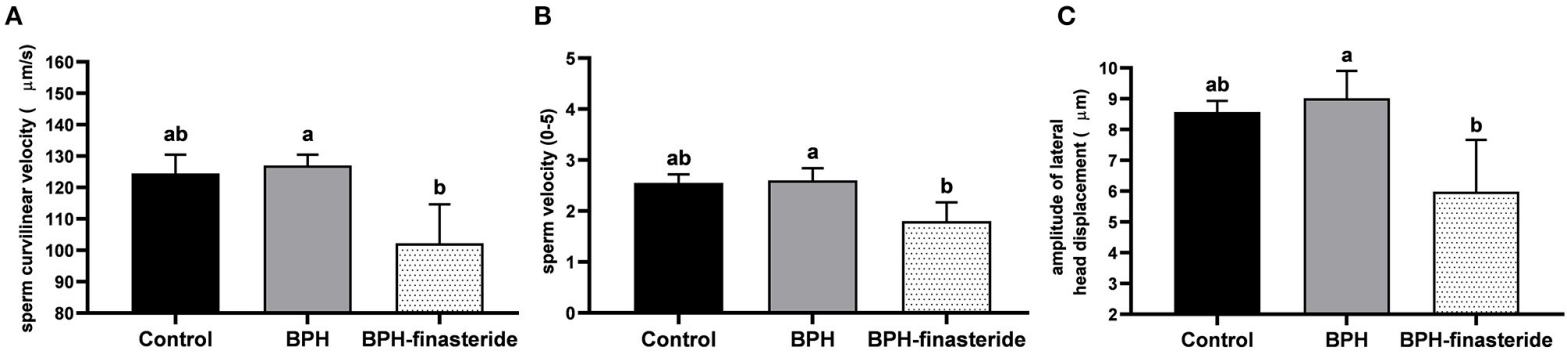
Mean and standard error of the post-thawed **(A)** sperm curvilinear velocity (μm/s) **(B)** sperm velocity (0–5) and **(C)** sperm amplitude of lateral head displacement (μm) in the Control, BPH and BPH-Finasteride groups. ^a−b^ indicate significant difference (*P* < 0.05) among experimental groups.

In relation to the post-thawed samples, sperm curvilinear velocity was higher in the BPH group compared to the BPH-Finasteride group (Figure 3A), so as sperm velocity (Figure 3B) and amplitude of lateral head displacement (Figure 3C).

**Figure 4:**
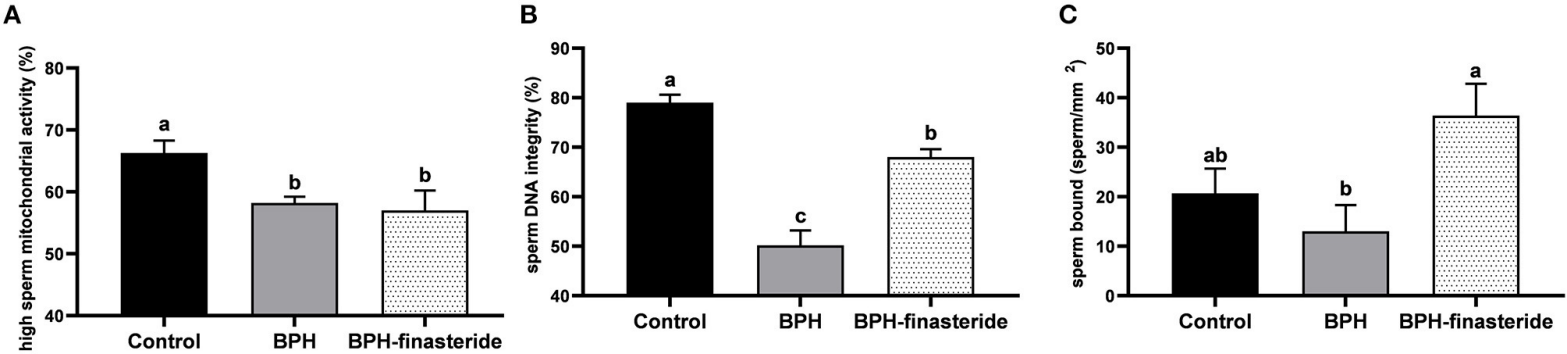
Mean and standard error of the post-thawed **(A)** high sperm mitochondrial activity (%); **(B)** sperm DNA integrity (%) and **(C)** sperm count bound to perivitelline membrane (sperm/ mm2) in the Control, BPH and BPH-Finasteride groups. ^a−b^ indicate significant difference (*P* < 0.05) among experimental groups.

The control group had the greatest percentage of high sperm mitochondrial activity in comparison to BPH and BPH-Finasteride dogs (Figure 4A). In addition, sperm DNA integrity rate was highest in the Control group, followed by BPH-Finasteride dogs (Figure 4B). BPH dogs had the lowest post-thawed sperm DNA integrity (Figure 4B). The analysis of sperm bound to perivitelline membrane showed that BPH dogs had lower sperm count in comparison to BPH-Finasteride group (Figure 4C).

No differences among groups were verified for the remaining seminal variables, both in fresh and post-thawed sperm samples (Tables 1, 2, respectively).

**Table 1:** Mean and standard error (X ± SEM) of the fresh sperm analysis in control, BPH and BPH-finasteride groups.

**Variable**	**Control**	**BPH**	**BPH-finasteride**
Volume (mL)	1.5 ± 0.6	0.7 ± 0.2	0.6 ± 0.1
Sperm concentration (x10^6^/mL)	336.6 ± 70.5	535 ± 252.7	160.8 ± 60.8
Sperm concentration per ejaculate (x10^6^)	357.05 ± 83.1	264.5 ± 107.8	118.17 ± 52.9
Plasma membrane integrity (%)	86.6 ± 2.5	78.4 ± 6.3	82.16 ± 4
Acrosome integrity (%)	98.3 ± 0.5	95 ± 1.5	97 ± 1.4
Major sperm defects (%)	11.8 ± 3.2	20.4 ± 4.3	19.3 ± 4.4
Minor sperm defects (%)	4.6 ± 1.1	3.6 ± 1.6	3.8 ± 1.1
Total sperm defects (%)	29.8 ± 8.7	24 ± 5.0	23.1 ± 4.6
High mitochondrial activity (%)	71.3 ± 1.0	66.4 ± 12	73.3 ± 6.7
Medium mitochondrial activity (%)	18.6 ± 1.1	10.4 ± 1.7	6.6 ± 2.6
Low mitochondrial activity (%)	6.1 ± 0.7	3.8 ± 1.1	4.1 ± 1.4
Absent mitochondrial activity (%)	4.1 ± 0.9	19.4 ± 13.7	12.5 ± 5.2
Sperm count bound to perivitelline membrane (sperm/ mm^2^)	97.3 ± 25.7	52.8 ± 28.0	36.8 ± 14.7
TBARS (ng/10^6^ spermatozoa)	867.9 ± 260.1	1,795.3 ± 710.5	1,367.4 ± 221.9
Sperm progressive motility (%)	34.1 ± 5	27 ± 8.5	44.1 ± 10.6
Rapid sperm velocity (%)	41.6 ± 5	33.6 ± 9.7	49.6 ± 12.1
Sperm straightness (%)	83.7 ± 2.8	82 ± 7.8	87.8 ± 3.1
Sperm linearity (%)	59.4 ± 5.8	60.2 ± 13.5	64.8 ± 5.9
Sperm VAP (μm/s)	101.9 ± 3.5	98.9 ± 14.3	116.9 ± 10.1
Sperm VSL (μm/s)	86.1 ± 4.7	83.4 ± 14.2	104.4 ± 10.2
Sperm VCL (μm/s)	155.5 ± 11.7	141 ± 17.6	165 ± 11.1
Sperm ALH (μm/s)	6.8 ± 0.7	6.3 ± 0.8	6.6 ± 0.8
Static Sperm (%)	14.6 ± 5.0	14 ± 5.1	20.6 ± 7
Intact plasma and acrosome membrane (%)	83.2 ± 1.6	63.5 ± 20.4	76.7 ± 5.5
Plasma membrane injury (%)	10.5 ± 0.6	21.6 ± 11.5	11.2 ± 2.8
Acrosome membrane injury (%)	2.1 ± 0.4	2.6 ± 1.2	4.07 ± 0.9
Plasma and acrosome membrane injury (%)	4.03 ± 0.9	12.1 ± 7.6	7.9 ± 2.4
High mitochondrial potential (%)	58.2 ± 8.8	40.4 ± 6.5	50.9 ± 14.7
Intermediate mitochondrial potential (%)	27.2 ± 7.2	30.2 ± 5	26.2 ± 8.2

**Table 2:** Mean and standard error (X ± SEM) of the post-thaw sperm analysis in Control, BPH and BPH-Finasteride groups.

**Variable**	**Control**	**BPH**	**BPH-Finasteride**
Plasma membrane integrity (%)	55.8 ± 4.2	47 ± 6.3	40.8 ± 8.3
Acrosome integrity (%)	95.6 ± 0.7	92.8 ± 2.1	93.6 ± 1.5
Major sperm defects (%)	48 ± 8.1	41.4 ± 6	49.6 ± 6.2
Minor sperm defects (%)	9.5 ± 1.5	7.6 ± 1.7	8.8 ± 1.6
Total sperm defects (%)	57.5 ± 7.2	49 ± 4.9	58.4 ± 7.1
Medium mitochondrial activity (%)	23.4 ± 2.4	30 ± 1.9	28.6 ± 5
Low mitochondrial activity (%)	5.5 ± 1.5	7.8 ± 3.1	6.8 ± 3.3
Absent mitochondrial activity (%)	5.1 ± 0.8	6.2 ± 1.5	7.8 ± 1.9
TBARS (ng/10^6^ spermatozoa)	2,461.4 ± 277.1	1,907.6 ± 140.3	2,042.3 ± 191.7
Total sperm motility (%)	30.5 ± 4.5	18 ± 7.1	22 ± 7.3
Progressive sperm motility (%)	3.2 ± 1.2	2.2 ± 1.2	9.2 ± 6.1
Rapid sperm velocity (%)	4 ± 1.4	2.2 ± 1.2	10.6 ± 7.3
Medium sperm velocity (%)	25 ± 4.8	14 ± 6.6	11.6 ± 1.6
Slow sperm velocity (%)	53.1 ± 4.9	41.6 ± 11	51.4 ± 13.7
Sperm straightness (%)	81.7 ± 2.9	85.6 ± 3.1	76.8 ± 7.7
Sperm linearity (%)	48.7 ± 1	59.4 ± 7.9	49.8 ± 7.1
Sperm VAP (μm/s)	70.9 ± 3.2	83 ± 11.6	62.1 ± 9.7
Sperm VSL (μm/s)	58.2 ± 2.5	73.1 ± 13.5	50.9 ± 10.7
Sperm VCL (μm/s)	124.5 ± 5.9	127 ± 3.4	102.2 ± 12.4
Sperm BCF (Hz)	22.9 ± 1	18.9 ± 4.5	27.1 ± 3.6
Sperm ALH (μm/s)	8.5 ± 0.3	9 ± 0.8	5.9 ± 1.6
Static Sperm (%)	17.8 ± 6.3	42.2 ± 17.5	26.6 ± 12.9
Intact plasma and acrosome membrane (%)	30.8 ± 5.9	23.2 ± 9.6	28.8 ± 4.0
Plasma membrane injury (%)	38.6 ± 2.9	38.9 ± 9.5	33.4 ± 3.4
Acrosome membrane injury (%)	0.7 ± 0.1	4.6 ± 3.6	1.4 ± 0.2
Plasma and acrosome membrane injury (%)	29.6 ± 3.6	33.1 ± 3.7	36.1 ± 4.5
High mitochondrial potential (%)	60.3 ± 8.5	59.9 ± 7.5	57.1 ± 10.9
Intermediate mitochondrial potential (%)	24.8 ± 6.7	26.6 ± 5.9	29.1 ± 6.4
Low mitochondrial potential (%)	14.8 ± 2.3	13.4 ± 2.2	13.7 ± 6.5

## Discussion

In the present study, sperm freezability of BPH and BPH-finasteride treated dogs was compared to control dogs, as to identify any possible influence of the prostatic disease and its most frequent medical treatment on post-thaw semen samples. Benign Prostatic Hyperplasia is highlighted as the most important male reproductive disease in senility, and negatively affects reproductive potential and sperm features in dogs (3, 4, 26). However, to our knowledge, no studies have been performed in order to describe the relationship between BPH seminal alterations and cryoinjury.

In regards to the fresh semen evaluation, BPH-Finasteride group had the lowest ejaculate visual aspect of opacity, which can be interpreted indirectly by lowering of sperm concentration of the ejaculate, compared to control and BPH dogs. On the other hand, we could not observe ejaculate volume and total sperm count differences among groups after 60 consecutive days of finasteride treatment in the present research, nor within previous studies (2). Thus, it is possible to hypothesize that finasteride therapy altered the appearance of the prostatic fluid, making it difficult to perform a visual inspection of the ejaculate aspect (opacity). Additionally, the altered biochemical composition of the prostatic fluid of BPH dogs, in regards to cholesterol, pH, osmolarity and mineral content, may influence fluid appearance (5). In fact, finasteride treatment is capable of diminishing to 46% the androgen receptor expression in prostatic cells, thus reducing androgenic influence on prostate secretion (27). As an important side-effect of finasteride, lowering of libido has also been attributed to medically treated BPH patients (28), thus, we can suggest that a diminished sperm output during sperm collection through manual stimulation is the cause under the low ejaculate aspect of BPH-Finasteride group. Thus, based on the present findings, it is also advisable not to employ the visual analysis of viscosity of the ejaculate as manner to attest sperm count in both HPB and finasteride-treated dogs.

Despite the absence of alterations in sperm count, finasteride treatment promoted lowest values of fresh sperm total motility and percentage of medium velocity, as well as lowest post-thaw sperm curvilinear velocity and amplitude of lateral head displacement. Therefore, we can also suggest that finasteride therapy alters sperm kinetics, compromising sperm fertilizing capability, even of post-thaw semen samples. In addition, fresh sperm flagellar beating frequency (BCF) was higher in finasteride treated dogs, in comparison to control dogs, suggesting a kinetic pattern similar to premature sperm hyperactivation (29). In general, large amount of reactive oxygen species (ROS) generation, modifications in pH and osmolarity of the prostatic fluid are responsible in promoting sperm premature hyperactivation (30). In the present study, since no differences in oxidative stress were verified among the experimental groups, it is possible to infer that sperm kinetics alterations may occur as an influence of the prostatic fluid disruption itself rather than the negative effects of ROS. However, future studies focused on the analysis of the seminal plasma biochemical content should be performed to confirm this hypothesis. In addition, a direct influence of finasteride on spermatogenesis cannot be ruled out. Indeed, Chen et al. (31) showed significantly up-regulation of testicular pre-apoptotic genes and apoptosis of spermatogonia, Sertoli and Leydig cells, thus leading to degenerative spermatozoa in seminiferous tubules of finasteride treated HPB-rats. Hence, finasteride treatment induces spermatogenic disruption, which contributed to alterations of fresh sperm kinetics that persisted after cryopreservation.

Fresh semen samples of the BPH group had low sperm mitochondrial potential in addition to higher rates of sperm slow velocity, in compared to control dogs. Sperm mitochondria are the main energy source for motility, chemical homeostasis and sperm metabolism (32), which is highly related to fertility rate (26). Taken into account both results, it is reasonable to suggest a reduction in sperm metabolism, which can be considered a direct consequence of both senility and BPH on spermatogenesis (3, 33). The etiological hormonal imbalance of BPH can directly affect spermatogenesis, since testosterone synthesis is essential for spermatozoa production (2, 8). Spermatogenic injuries through hormonal imbalance can also affect sperm DNA integrity, ultimately compromising sperm function (2, 10). In fact, BPH dogs had the lowest percentage of sperm DNA integrity in both fresh and post-thawed samples. Thus, we can suggest that the primary endocrine disruption of BPH is the main cause under sperm morpho-functional defects, such as altered kinetics and DNA structure.

Post-thawed sperm of the BPH and BPH-finasteride groups had lower sperm mitochondrial activity, suggesting a more marked reduction in energetic activity of BPH sperm subjected to cryoinjury. On the other hand, post-thawed sperm of finasteride treated dogs had lower motility parameters, compared to untreated group (BPH), such as, sperm curvilinear velocity, sperm velocity and amplitude of lateral head displacement. Again, the related negative influence of finasteride on testicular spermatogenesis can account for an altered sperm motility pattern in fresh semen that worsens after cryopreservation. Conversely, finasteride treatment had a beneficial influence on the number of post-thawed spermatozoa bound to egg yolk perivitelline membrane and the pertecentage of sperm DNA integrity, in comparison to BPH dogs. Interestingly, finasteride treatment had no influence on fresh sperm DNA integrity and penetration capability, similarly to the results shown by Angrimani et al. (33). Hence, we can assume that finasteride treatment diminished sperm DNA susceptibility to cryodamage in HPB dogs. However, the exact mechanism by which finasteride favors sperm DNA integrity after cryopreservation remains unexplained, although Angrimani et al. (33) suggested that prostatic fluid zinc deficiency of HPB dogs accounts for higher sperm DNA damage. Finasteride treatment can possible restore the biochemical content of the prostatic fluid, thus neutralizing the inherent susceptibility of sperm DNA to injuries of cryopreservation in HPB dogs. Taking together such results, finasteride treatment appears to play a positive role in sperm cryopreservation, compared to untreated BPH dogs, favoring sperm fertilizing capacity (34).

## Conclusion

In conclusion, BPH modifies sperm features, which in turn alters spermatozoa freezability, in special reference to mitochondrial and sperm DNA integrity. Thus, the ejaculate of BPH dogs has higher susceptibility to cryoinjury. Albeit BPH dogs treated with finasteride have lowered post-thaw sperm kinectics, spermatozoa functional performance (sperm bound test) increased, suggesting a promising use of BPH dogs as semen donors for sperm cryopreservation programs.

## Data Availability Statement

The raw data supporting the conclusions of this article will be made available by the authors, without undue reservation.

## Ethics Statement

The animal study was reviewed and approved by Bioethics Committee of the School of Veterinary Medicine and Animal Science – University of São Paulo. Written informed consent was obtained from the owners for the participation of their animals in this study.

## Author Contributions

Conceptualization and design of the study: RBF and CIV. Methodology, acquisition of data, analysis, and interpretation of data: RBF, MMB, LLA, JVML, and JDAL. Writing—original draft preparation: RBF, DSRA, and CIV. Revision of the manuscript critically for important intellectual content and final approval of the version to be submitted: CIV. All authors contributed to the article and approved the submitted version.

## Funding

This work was supported by FAPESP, Grant Number 2017/05492-0.

## Conflict of Interest

The authors declare that the research was conducted in the absence of any commercial or financial relationships that could be construed as a potential conflict of interest.

## Publisher's Note

All claims expressed in this article are solely those of the authors and do not necessarily represent those of their affiliated organizations, or those of the publisher, the editors and the reviewers. Any product that may be evaluated in this article, or claim that may be made by its manufacturer, is not guaranteed or endorsed by the publisher.
